# Hypertension and age‐related focal global glomerulosclerosis are associated with biomarkers for cellular senescence

**DOI:** 10.14814/phy2.70730

**Published:** 2026-01-29

**Authors:** Michael D. Hughson, Alaa A. Ali, Yusuke Okabayashi, Victor G. Puelles, John F. Bertram

**Affiliations:** ^1^ Hiwa Oncology and Shoresh General Teaching Hospitals Sulaimania Iraq; ^2^ University of Mississippi Medical Center Jackson Mississippi USA; ^3^ III. Department of Medicine University Medical Center Hamburg‐Eppendorf Hamburg Germany; ^4^ Division of Nephrology and Hypertension, Department of Internal Medicine The Jikei University School of Medicine Tokyo Japan; ^5^ Department of Clinical Medicine Aarhus University Aarhus Denmark; ^6^ Department of Pathology Aarhus University Hospital Aarhus Denmark; ^7^ Hamburg Center for Kidney Health Hamburg Germany; ^8^ Department of Anatomy and Developmental Biology, Biomedical Discovery Institute Monash University Melbourne Victoria Australia; ^9^ ARC Training Centre for Cell and Tissue Engineering Technologies Monash University Melbourne Victoria Australia

**Keywords:** arterionephrosclerosis, focal global glomerulosclerosis, glomerulosclerosis, hypertension

## Abstract

Arterionephrosclerosis is characterized by focal global glomerulosclerosis (FGGS), which is a constant feature of aging and hypertension. FGGS begins as normal‐appearing glomeruli that undergo tuft contraction (TC) and progress to global glomerulosclerosis (GGS). Kidney tissue from 26 hypertensive and 25 age‐matched non‐hypertensive patients was analyzed for glomerular volume and for podocyte number using a WT1 antibody. Immunohistochemistry (IHC) was employed to detect the senescence‐related biomarkers p16, p21, β‐galactosidase (GLB1), and 5‐nucleotidase (CD73). Antibodies against annexin 3 (ANXA3), cytokeratin 7, and CD44 were used to evaluate parietal epithelial cell (PEC) activation. The relationships between biomarkers, hypertension, TC, and GGS were quantitatively analyzed. With TC, podocyte numbers decreased in association with increased glomerular p16, p21, GLB1, and CD73 expression. With TC, WT1, CK7, and CD44‐expressing PEC increased. TC and GGS expressed senescent markers in hypertensive and non‐hypertensive kidneys; however, the frequency of TC (*p* < 0.01) and GGS (*p* < 0.001) was greater in hypertensive kidneys, and glomerular expression of senescence markers was correspondingly higher. Additionally, greater p16 and p21 expression was observed in the tubular atrophy of hypertension. As FGGS developed, podocyte depletion, cellular senescence markers, and PEC activation were associated with TC and increased with hypertension.

## 1 INTRODUCTION

Chronic hypertension is characterized by intrarenal arteriosclerosis, interstitial fibrosis and tubular atrophy (IF/TA), and glomerulosclerosis (Hall et al., 2012; Hughson et al., 2014; Shankland et al., 2023). The glomerulosclerosis of hypertension is different from that of primary or secondary glomerular diseases and is referred to as glomerular ischemia, glomerular obsolescence, or focal global glomerulosclerosis (FGGS). FGGS is generally preferred because it does not presume a cause (Denic, Lieske, et al., 2017; Hommos et al., 2017; Hughson et al., 2003, 2014).

FGGS undergoes a sequence of changes that are thought to begin with glomerular tuft contraction. The glomerular capillary basement membranes of the contracting tuft become thickened and wrinkled, and the capillaries are simplified into fewer lobules as the tuft shrinks toward the vascular pole (Denic, Lieske, et al., 2017; Hommos et al., 2017; Hughson et al., 2002). Parietal epithelial cells (PEC) become prominent, and poorly cellular collagen accumulates in Bowman's space, surrounding the contracting tuft within a largely intact Bowman's capsular basement membrane. The late stage of FSGS, which we will refer to as global glomerulosclerosis (GGS), consists of a brightly PAS‐positive hyalinized nodule that eventually disappears from the kidney (Denic, Lieske, et al., 2017; Hughson et al., 2002).

Understanding the pathogenesis of FGGS is important because hypertension is second only to diabetes as a cause of end‐stage kidney disease (ESKD) (Burrows et al., 2022). Recent studies have attributed glomerular loss in hypertension to primary podocyte depletion, possibly related to premature glomerular senescence (Hodgin et al., 2015; Puelles et al., 2016; Shankland et al., 2023; Wang et al., 2009). In several studies, the “normal” appearing glomeruli in hypertensive subjects are reported to be larger than those in subjects without hypertension (Denic et al., 2018; Hodgin et al., 2015; Hughson et al., 2014; Puelles et al., 2016).

Glomerular enlargement likely reflects a compensatory response to the increased blood flow required to maintain cardiac output against the elevated peripheral vascular resistance associated with hypertension (Hall et al., 2012), but the mechanisms and contributions of this response to FGGS remain uncertain. To investigate the structural changes associated with FGGS in hypertensive and non‐hypertensive aging patients, we evaluated the expression of senescence biomarkers, podocyte depletion, and PEC activation across the phases of “normal” glomeruli, tuft contraction (TC), and GGS.

## 2 METHODS

The research used human tissue consisting of 36 biopsies and normal renal tissue from 15 nephrectomies and was performed according to the Helsinki Accords. Biopsies were considered satisfactory if they contained eight measurable glomeruli and 4 “normal‐appearing” glomeruli within the tissue core. Normal renal tissue from nephrectomies for neoplastic disease was obtained distant from the tumor and cut into 3–4 mm wide cores containing the full thickness of the cortex with 38–52 glomeruli. The Scientific Research Unit of Hiwa Hematology and Oncology Hospital approved the use of the tissues as exempt from informed consent. Tissue samples were obtained for diagnostic purposes, with all material stored as paraffin blocks in departmental archives. No additional patient testing was performed.

### 2.1 Blood pressure assessment and assignment of hypertension

Blood pressure, serum creatinine, and a clinical diagnosis of obesity were obtained from medical records. Estimated GFR (eGFR) was derived from the NKF interactive website using the 2021 CKD EPI calculation. Subjects were categorized as hypertensive if blood pressure was ≥140/90 mmHg or if there was a history of and treatment for hypertension.

### 2.2 Morphometric analysis

Blocks sectioned at 3 μm thickness were stained with hematoxylin and eosin (H&E), Masson's trichrome, periodic‐acid Schiff‐hematoxylin (PAS‐H), and periodic‐acid methenamine silver stains. The frequency of TC and GGS, and the extent of IF/TA and arterial intimal thickening were determined using PAS‐H‐stained sections. Arteriolosclerosis was measured in interlobular arteries 90–250 μm in diameter as the linear thickness of the intima to the outer arterial wall. IF/TA was measured by point counting as the proportion of blue‐staining cortex with a Masson trichrome stain.

### 2.3 Assessment of glomerular podocyte density and number and kidney senescence markers in biopsies of hypertensive and non‐hypertensive patients

Tissue from 26 patients with a diagnosis of primary hypertension consisted of 25 biopsies and one nephrectomy specimen. The hypertensive patients were biopsied because of an unusually severe or difficult‐to‐control elevation of blood pressure. Tissue from non‐hypertensive patients consisted of 11 biopsies with “normal kidney tissue” and 14 nephrectomies. The clinical reasons for obtaining non‐hypertensive kidney biopsies were mild (<2.5 gm/24 h) unexplained proteinuria, 5 biopsies, and donor biopsies for transplant kidney dysfunction in the first week post‐transplantation, 6 biopsies. In hypertensive patients, secondary hypertension was evaluated by abdominal ultrasound for kidney and adrenal gland size, Doppler renal blood flow studies, and plasma renin and aldosterone levels.

In addition to standard stains, 15 consecutive 3 μm sections were fixed on charged slides for immunohistochemistry (IHC) and stained in sequence with the antibodies listed below. The thickness of sections for IHC was checked at 1000× under oil by measuring the distance between the upper and lower surfaces of glomeruli using the calibrations on the high‐magnification focusing dial.

The following anti‐human antibodies with dilutions were used: anti‐WT1, 1:150 (Invitrogen, catalogue no. MA5‐38406, Thermo Fisher, Rockford, IL), anti‐p16, prediluted (P16ink4a, Bio SB clone RM‐67, catalogue no. BSB‐3768‐7, Santa Barbara, CA); anti‐p21, 1:150 (Invitrogen, catalogue no. MA5‐42680, Thermo Fisher, Rockford, IL); anti‐β‐galactosidase, 1:500 (GLB1, Invitrogen, catalogue no. PA5‐85195, Thermo Fisher, Rockford, IL); anti‐5‐nucleotidase, 1:200 (anti‐CD73, Invitrogen, catalogue no. MA5‐38673, Thermo Fisher, Rockford, IL); anti‐p53, 1:250 (Clone: D07, catalogue no. BSB‐5845, Bio SB, Santa Barbara, CA); and anti‐Ki‐67, 1:100 (Clone MM1, catalogue no. NCL‐L‐Ki67‐MM1, Leica Biosystems, Newcastle Upon Tyne, UK). The IHC detection system employed Polink‐2 Plus (OriGene, Catalogue no. D41‐110, Rockville, MD) with horseradish peroxidase and di‐amino‐benzidine (DAB).

The glomerular tuft area and podocytes per tuft profile were analyzed with an Olympus DP71 camera and imaging system at 400× magnification, with images viewed full‐screen. An orthogonal grid, imported onto the screen and calibrated with an ocular micrometer, had test points covering an area of 0.0004 mm^2^ at 400×. Biopsy glomerular tuft volume was estimated on PAS‐H‐stained sections by the Weibel and Gomez method (Weibel & Gomez, 1962) with the formula: glomerular volume = glomerular profile area^1.5^ × 1.38/1.01, where 1.38 is the value of a sphere, and 1.01 is a coefficient assuming a size variation of 10%.

Visceral podocytes identified as DAB‐stained brown nuclear profiles within or on the outer surface of the glomerular tuft were counted with the particle analyzing function of Image ProPlus 6.0 (Media Cybernetics, Rockville, MD) morphometric software. As described by Venkatareddy et al. (2014), podocyte density (per mm^3^) was determined as the number of WT1‐positive nuclei in a glomerular cross‐section area (μm^2^) times a human correction factor of 0.27 for a “true podocyte number” times three for the section thickness, with the result being podocyte density in a 3‐dimensional profile (μm^3^). The number of podocytes per glomerular tuft for a biopsy was calculated by multiplying the average podocyte density by the average glomerular tuft volume.

GLB1 and CD73 were quantified as the proportional area of staining in the cross‐sections of “normal” glomeruli, glomeruli with TC, and GGS. Total GLB1 and CD73 cross‐sectional area staining was tabulated for each glomerular pattern. Glomerular p16 and p21 were quantified as the number of stained cells in the cross‐sections of “normal” glomeruli, glomeruli with TC, and GGS. p16, p21, and Ki‐67 tubular staining was estimated as the number of positive cells in five 400× microscopic fields (1.2 mm^2^) of cortex. Vascular staining for p21 was estimated as the proportion of positive cells in a cross‐section of a 90–250 μm interlobular artery. Vascular p16 staining was evaluated as positive or negative.

### 2.4 Parietal epithelial cell activation

Immunofluorescence staining was performed on formalin‐fixed paraffin‐embedded biopsy sections to evaluate the role of parietal epithelial cells (PECs) in the development of FGGS. A total of five samples from both hypertensive and non‐hypertensive groups were stained. Cytokeratin 7 (CK7) and CD44 immunofluorescence were performed using mouse monoclonal anti‐CK7 antibody (1:10 dilution; GA61961‐2, Agilent, Santa Clara, CA) and mouse monoclonal anti‐CD44 antibody (1:200 dilution, 5640S, Cell Signaling Technology, Danvers, MA), respectively. Each procedure included co‐staining with anti‐annexin A3 (anti‐ANXA3) (1:200 dilution, HPA013398, Sigma‐Aldrich, St Louis, MO) as a PEC marker and DAPI (1:200 dilution, D9542, Sigma‐Aldrich) for nuclei. Secondary antibodies consisted of donkey anti‐mouse IgG Alexa Fluor 647 (1:200 dilution, Thermo Fisher Scientific, A31571) and donkey anti‐rabbit IgG Alexa Fluor 555 (1:200 dilution, Thermo Fisher Scientific, A31572). Sections were evaluated using a confocal microscope with AryScan (Celldiscover 7) and Keyence BZ‐IIZEN 3.7 software (Carl Zeiss AG, Germany).

### 2.5 Statistical methods

Analyses were performed with Stata/IC 10.0 (StataCorp Statistical Software, StataCorp LP, College Station, Texas). Two‐way comparisons were performed by a *t*‐test if variables passed normality and equal distribution tests or by Wilcoxon rank sum tests if they did not. Multivariable analyses used least‐squares regressions. Values are presented as mean ± standard deviation. A *p* value <0.05 was considered statistically significant.

## 3 RESULTS

### 3.1 Demographics and histopathology

The biopsy and nephrectomy tissue were from Iraqi Kurds, a Caucasian population with genetic links to Iranians and Armenians. The clinical and pathological data are summarized in Table 1.

**TABLE 1:** Clinical and pathologic characteristics of patients having biopsies of hypertensive nephrosclerosis and non‐hypertensive “normal” kidneys.

Characteristic	Hypertension (*n* = 26)	Non‐hypertension (*n* = 25)	*p* Value
Age (years)	49.0 ± 10.1	47.4 ± 10.6	0.57
Male	0.62	0.72	0.62
Systolic blood pressure, mmHg	166.5 ± 22.0	124.3 ± 5.5	<0.001
Diastolic blood pressure, mmHg	104.6 ± 12.3	78.2 ± 4.4	<0.001
Serum creatinine	1.53 ± 0.60	1.04 ± 0.05	0.002
eGFR	76 ± 26	97 ± 23	0.007
Obese	None	1	—
GGS (proportion)	0.20 ± 0.15	0.03 ± 0.04	<0.001
TC (proportion)	0.14 ± 0.03	0.03 ± 0.03	0.002
Arterial It (ratio)	0.16 ± 0.10	0.03 ± 0.03	<0.001
IF/TA (proportion)	0.26 ± 0.21	0.03 ± 0.03	<0.001

Abbreviations: eGFR, estimated glomerular filtration rate (CKD‐EPI, creatinine, www.kidney.org>kdoqi>gfr_calculator); GGS, global glomerulosclerosis; IF/TA, interstitial fibrosis and tubular atrophy; TC, tuft contraction; arterial It, arterial intimal thickening.

**FIGURE 1:**
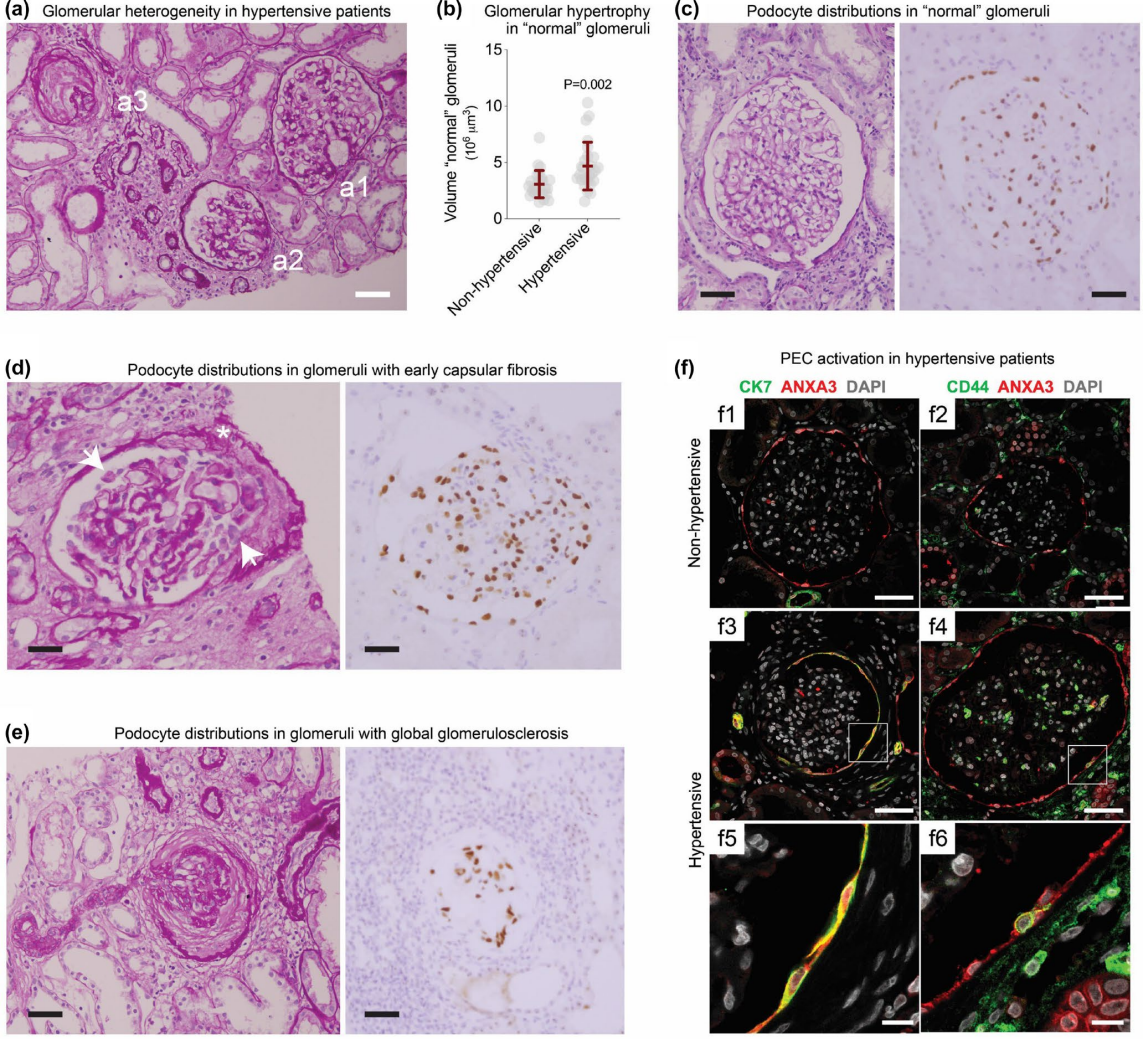
(a) Histologic features of arteriolonephrosclerosis, showing glomeruli in the different stages of focal global glomerulosclerosis (FGGS). (a1) A “normal” glomerulus in a hypertensive kidney biopsy. (a2) A glomerulus with tuft contraction and capsular fibrosis. (a3) A globally sclerotic glomerulus with intracapsular fibrosis enveloping a completely contracted tuft. Periodic acid‐Schiff‐hematoxylin (PAS‐H) stain, scale bar 100 μm. (b) The volumes of “normal”‐ appearing glomeruli (μm^3^ × 10^6^) in hypertensive and non‐hypertensive kidneys differ significantly (*p* = 0.002), with glomeruli in hypertensive subjects skewed toward very large sizes. The interquartile range and median volume of “normal” appearing glomeruli (non‐hypertension: 2.32–2.65–3.51 μm^3^ × 10^6^; hypertension: 3.39–4.14–5.50 μm^3^ × 10^6^) shows an overlap in the lower and middle quartiles. (c) A “normal” glomerulus in a hypertensive patient (PAS‐H stain). WT1 staining shows a predominance of small, flattened nuclear profiles, with relatively few larger, oval nuclei. Scale bars 50 μm. (d) A Glomerulus showing capsular fibrosis and glomerular tuft contraction (PAS‐H stain). The glomerulus demonstrates simplification and contraction of the tuft with intracapsular fibrosis at the tubular pole (asterisk). Dehisced epithelial cells (arrows) are seen in Bowman's space, free from glomerular or capsular basement membranes. WT1 staining shows larger nuclear contours than those of “normal” glomeruli. WT1‐positive parietal epithelial cells are prominent. Scale bars 50 μm. (e) PAS‐H stain of the late stage of global glomerulosclerosis consists of a hyaline tuft containing cells that are WT1‐positive and consistent with altered podocytes. WT1‐positive cells are also present within the intracapsular fibrosis, consistent with entrapped parietal epithelial cells. Scale bars 50 μm. (f) Representative images of ANEXIN 3, cytokeratin 7, and CD44 staining in non‐hypertensive (f1, f2) and hypertensive patients (f3, f4, f5, and f6). Bowman's capsule of non‐hypertensive patients is lined by flat parietal epithelial cells (PECs) showing only red annexin A3 (ANXA3) staining. In hypertensive kidneys, increased numbers of cells lining Bowman's capsule show the green fluorescence of CK7 and CD44. Scale bars: (f1–f4) 50 μm, (f5 and f6) 10 μm.

There was no significant difference in the age or sex distribution of hypertensive and non‐hypertensive patients (age, *p* = 0.59; sex, *p* = 0.62). The blood pressure of hypertensive patients averaged well into the moderately hypertensive range, and three patients had severe hypertension. TC, GGS, arteriolosclerosis, IF/TA (Figure 1), and serum creatinine were significantly higher in hypertensive patients than in non‐hypertensive patients. eGFR was decreased with hypertension. None of the hypertensive patients and one of the non‐hypertensive patients were obese.

All of the glomerulosclerosis in our material consisted of FGGS. Focal segmental glomerulosclerosis (FSGS) was not found, nor was there any glomerular solidification. The average volume of “normal” glomeruli was significantly larger in hypertensive patients than in non‐hypertensive patients (*p* = 0.002, Figure 1b). However, the “normal” glomeruli of hypertensive subjects were skewed toward unusually large sizes, with overlapping volumes that encompassed the median and lower quartiles of non‐hypertensive subjects. In multivariate regression, the larger volumes of the “normal” glomeruli were significantly associated with the proportion of GGS (coefficient 5.641, 95% CI, 1.162 to 10.119, *p* = 0.02) but not to age (coefficient 0.045, 95% CI, −0.015 to 0.105, *p* = 0.14), suggesting an adaptation for prior glomerular loss.

### 3.2 Podocyte density and number in biopsies of hypertension and non‐hypertensive patients

Table 2 summarizes the podometrics of the three categories of glomeruli in hypertensive and non‐hypertensive patients. The podocyte density of the larger “normal” glomeruli in hypertensive biopsies was less than in the “normal” glomeruli of non‐hypertensive biopsies, but the difference was not significant, and the “normal” glomeruli of hypertensive patients were estimated to have more podocytes than “normal” glomeruli in non‐hypertensive patients. As terminally differentiated cells, there is no reason podocyte numbers should increase in secondarily enlarged glomeruli. We believe the small, flat nuclear profiles in “normal” glomeruli were overestimated, possibly because the Weibel Gomez mathematical models (Weibel & Gomez, 1962) are sensitive to nuclear size and shape, as stated by Venkatareddy et al. (2014). In addition, podocyte numbers per tuft are computed from an average glomerular volume that, in hypertensive biopsies, was biased toward the very large “normal” glomerular volumes of some hypertensive subjects (Venkatareddy et al., 2014). When total podocyte numbers of “normal” glomeruli were adjusted by trimming the upper and lower 5% of the glomerular volumes, the total podocyte number of “normal” glomeruli of hypertensive subjects approached parity with that of non‐hypertensive subjects but was still significantly greater (*p* = 0.02).

**TABLE 2:** Comparisons of glomerular tuft volume, podocyte density, and podocytes per glomerulus in “normal” glomeruli, glomeruli with pericapsular fibrosis and tuft contraction, and global glomerulosclerosis of hypertensive and non‐hypertensive patients.

Glomerular pattern	Hypertension	Non‐hypertension	*p* Value
“Normal” glomeruli			
Volume (10^6^ μm^3^)	4.68 ± 2.13	3.07 ± 1.21	0.002
Podocyte density/tuft	149 ± 28	160 ± 23	0.11
Podocytes/tuft, adjusted^a^	619 ± 124	507 ± 175	0.02
TC			
Volume (10^6^ μm^3^)	1.93 ± 0.99	1.82 ± 0.73	0.73
Podocyte density/tuft	217 ± 91	141 ± 45	0.01
Podocytes/tuft	359 ± 146	238 ± 71	0.01
GGS			
Volume (10^6^ μm^3^)	1.3 ± 0.4	0.6 ± 0.4	0.13
Podocyte density/tuft	147 ± 119	122 ± 66	0.56
Podocytes/tuft	118 ± 78	71 ± 43	0.11

Abbreviations: GGS, global glomerulosclerosis; TC, tuft contraction.

^a^ Podocytes per tuft of “normal” glomeruli were adjusted by trimming the values from the upper and lower 5% of glomerular volumes for the skew toward large size in hypertensive subjects.

In glomerular patterns leading to GGS, glomerular volume and podocyte number decreased. There were qualitative and quantitative changes in podocytes in the change from “normal” glomeruli to TC. In “normal” glomeruli, the nuclei of WT1‐staining podocytes tended to have flattened oval shapes with small to medium‐sized nuclear profiles (Figure 1c). During TC, the nuclei of WT1‐staining podocytes assumed large spheroidal shapes. In “normal” glomeruli, WT1 staining of PEC nuclei was present but infrequent, and PECs were flat, whether WT1‐positive or WT1‐negative. During TC, PECs increased in number, became spheroidal, and many became WT1‐positive. Free cells unattached to Bowman's capsule or the glomerular tuft were frequently seen, but because of the PEC WT1 staining, it was difficult to discriminate podocytes from PECs (Figure 1d). With GGS, podocytes and WT1‐staining PECs were initially present (Figure 1e), but WT1 staining disappeared as the tuft became hyalinized.

### 3.3 Biomarkers of cellular senescence in biopsies of hypertension and non‐hypertension patients

A comparison of the staining of the markers p16, p21, Ki‐67, GLB1, and CD73 in hypertensive and non‐hypertensive biopsies is shown in Table 3.

**TABLE 3:** Quantitative expression of senescence markers and Ki‐67 in hypertensive and non‐hypertensive biopsies.

	Hypertensive biopsies			Non‐hypertensive biopsies			*p*
	Normal	TC	GGS	Normal	TC	GGS	
GLB1 glomerular area	0	0.641 ± 0.354	0.736 ± 0.417	0	0.239 ± 0.254	0.35 ± 0.3	−/0.001/0.10
CD73 glomerular area	0	0.837 ± 0.548	1.17 ± 0.72	0	0.429 ± 0.277	0.346 ± 0.409	−/0.01/0.001
p16+ cells per glomerulus	0.21 ± 0.39	2.35 ± 0.83	1.69 ± 0.70	0.15 ± 0.26	1.31 ± 0.79	1.95 ± 1.56	0.45/0.001/0.56
p16+ tubular cells/ 1.2 mm^2^	3.80 ± 3.45			1.01 ± 2.63			<0.001
p16+ arteries	Rare events 2 cases			2 cases			—
p21+ cells per glomerulus	1.76 ± 0.95	4.70 ± 2.92	3.26 ± 1.84	1.78 ± 0.97	3.17 ± 1.44	4.32 ± 2.47	0.92/0.09/0.31
p21+ tubular cells/ 1.2 mm^2^	95.3 ± 41.1			41.7 ± 18.4			<0.001
p21+ cells arteries	0.102 ± 0.047			0.055 ± 0.031			<0.01
Ki‐67+ tubular cells/1.2 mm^2^	29.3 ± 9.3			12.8 ± 3.7			<0.001

*Note*: *p* = significance of hypertension versus non‐hypertension; multiple *p* values for normal glomeruli/tuft contraction/GGS.

Abbreviations: GLB1, β‐galactosidase‐1; CD73, 5‐nucleotidase; TC, tuft contraction; GGS, global glomerulosclerosis.

**FIGURE 2:**
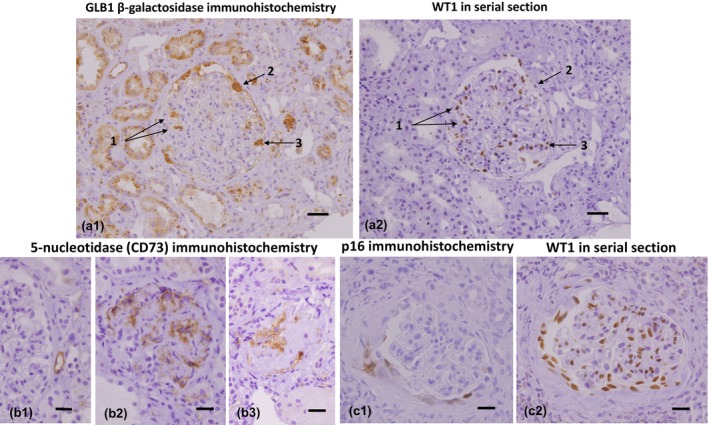
GLB1 β‐galactosidase and 5‐nucleotidase (CD73) immunohistochemistry. (a1) A hypertensive kidney demonstrates β‐galactosidase‐positive cells in a glomerulus with capsular fibrosis and early tuft contraction. (a2) In serial sections, the GLB1‐positive cells are matched with WT1‐positive podocytes and parietal epithelial cells. Arrows 1 point to intraglomerular tuft cells consistent with podocytes, arrow 2 to extraglomerular tuft cells consistent with parietal epithelial cells, and arrow 3 to an indeterminant cell. Scale bars 100 μm. (b) 5‐nucleotidase (CD73) immunohistochemistry. (b1) In a non‐hypertensive kidney, CD73 is found in interstitial capillaries but not glomerular capillaries. (b2) In a hypertensive kidney, CD73 staining is seen in a contracting glomerulus with capsular fibrosis. (b3) With global glomerulosclerosis, CD73 stains part of the hyalinized tuft and the surrounding intracapsular fibrosis. Scale bars: b1, 50 μm; b2, 35 μm; b3, 35 μm. (c) p16 immunohistochemistry. (c1) p16‐positive parietal epithelial cells are demonstrated in a contracting glomerulus of a hypertensive patient. (c2) A WT1‐stained serial section of the contracting glomerulus emphasizes that the p16‐stained cells are parietal epithelium and not intrinsic to the glomerular tuft. Scale bars: 50 μm.

GLB1 stained both podocytes and PECs, and with TC, the GLB1‐positive cells occupied a greater area of the glomeruli in hypertensive subjects than in non‐hypertensive subjects (Figure 2a). GLB1 disappeared as GGS developed.

CD73 (5′‐nucleotidase) was diffusely positive in interstitial but not glomerular capillaries in both hypertensive and non‐hypertensive kidneys. In hypertensive and non‐hypertensive kidneys, TC was accompanied by localized areas of glomerular basement membrane and podocyte CD73 staining (Figure 2b). As GGS developed, CD73 staining remained in the sclerotic tuft and the surrounding capsular fibrosis. In TC and GGS, glomerular CD73 staining was greater in hypertensive than in non‐hypertensive kidneys. GLB1 and CD73 were not found in the “normal” glomeruli of hypertensive or non‐hypertensive subjects.

“Normal” glomeruli rarely contained p16 or p21‐positive cells. p21 staining increased with TC and GGS in hypertensive and non‐hypertensive kidneys to nearly the same extent. Glomerular cells, only identified as PEC, were p16‐positive (Figure 2c). With TC, p16‐positive PEC were significantly increased in hypertensive kidneys.

**FIGURE 3:**
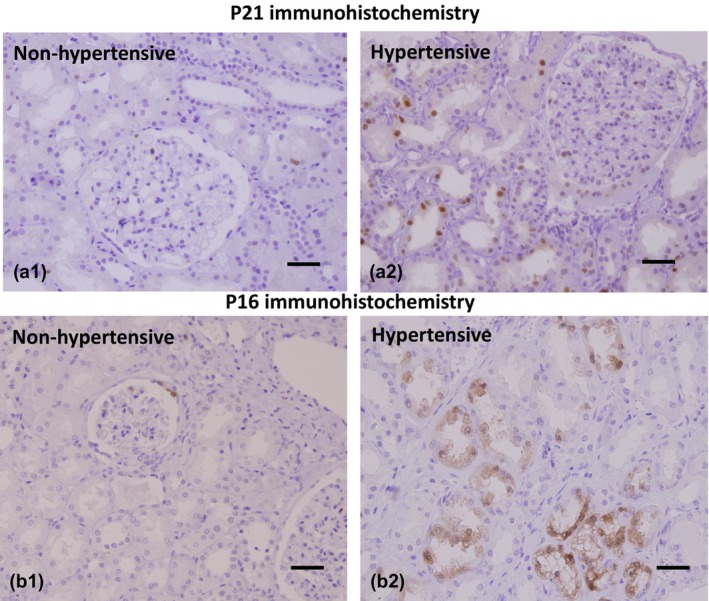
p21 and p16 immunohistochemistry. (a1) There is no p21 staining in a non‐hypertensive biopsy. (a2) A hypertensive kidney demonstrates 10%–15% staining of the tubular epithelium in an area of the cortex not involved by interstitial fibrosis or tubular atrophy. A “normal” glomerulus contains one or two P21‐positive cells. Scale bars 100 μm. (b1) A non‐hypertensive biopsy shows a contracting glomerulus with capsular fibrosis with two or three p16‐positive parietal epithelial cells as an internal control. There is no tubular p16 staining. (b2) A biopsy from a hypertensive patient demonstrates groups of proximal and distal tubules in an area of mild interstitial fibrosis, showing nuclear and cytoplasmic p16 staining. Scale bars 100 μm.

Staining for p16 and p21 was found in the epithelium of proximal and distal tubules (Figure 3a,b). The p16 and p21 staining tended to cluster in areas of IF/TA, but the number of p21‐positive cells/1.2 mm^2^ of cortex outside of the atrophic areas was significantly greater in hypertensive (95.3 ± 41.1) than non‐hypertensive (41.7 ± 18.4) kidneys, *p* < 0.001. p16 staining was more restricted to tubules in areas of IF/TA than p21, with the number of p16‐positive tubular cells being significantly greater in hypertensive kidneys, *p* < 0.001.

Smooth muscle and myointimal p21‐positive cells were proportionately greater in hypertensive specimens (*p* < 0.01) and were prominent in some arteries with marked intimal hyperplasia. Vascular p16 staining was a rare event found in small arteries that could not be quantified and involved equal numbers of hypertensive and non‐hypertensive biopsies. Low numbers of p53‐positive cells, with variable staining intensity, were observed in distal tubular cells, distributed in a pattern corresponding to p16 and p21. No p53 staining was found in glomeruli or arteries.

Ki‐67 expression was most frequently identified in distal tubular epithelium and was rare in glomeruli. The number of positive cells in 1.2 mm^2^ of the cortex was greater in hypertensive than non‐hypertensive kidneys (hypertension 29.3 ± 9.3 vs. 12.8 ± 3.7, *p* < 0.001). Ki67 had a weak direct correlation with p16 (coefficient 1.509, 95% CI, 0.0274–2.991, *p* = 0.046). It is not clear whether p16‐positive cells retained the ability to replicate or whether normal cells were replicating to replace senescent cells.

### 3.4 Parietal epithelial cell activation in biopsies of hypertensive and non‐hypertensive patients

**FIGURE 4:**
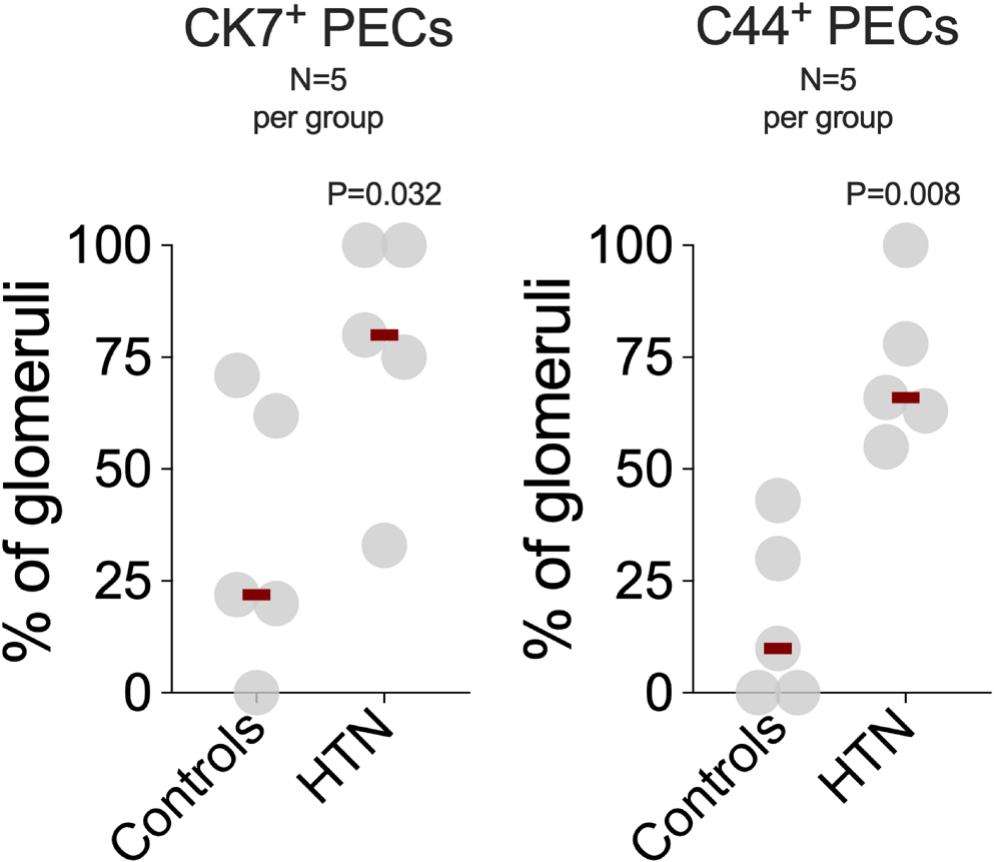
Immunofluorescent staining, measured in milliwatt units (MWU), shows a significant increase in CK7‐ and CD44‐positive parietal epithelial cells in hypertensive kidneys compared with non‐hypertensive control kidneys.

In non‐hypertensive biopsies, PECs were flat and ANXA3 positive, with minimal or no staining for CK7 or CD44 (Figure 1, f1 and f2). In hypertensive biopsies, CK7‐ and CD44‐positive PECs increased significantly compared with non‐hypertensive controls (Figure 1, f3–f6 and Figure 4).

### 3.5 Relationships between the glomerular expression of p16 and GLB1 with hypertension and age

**FIGURE 5:**
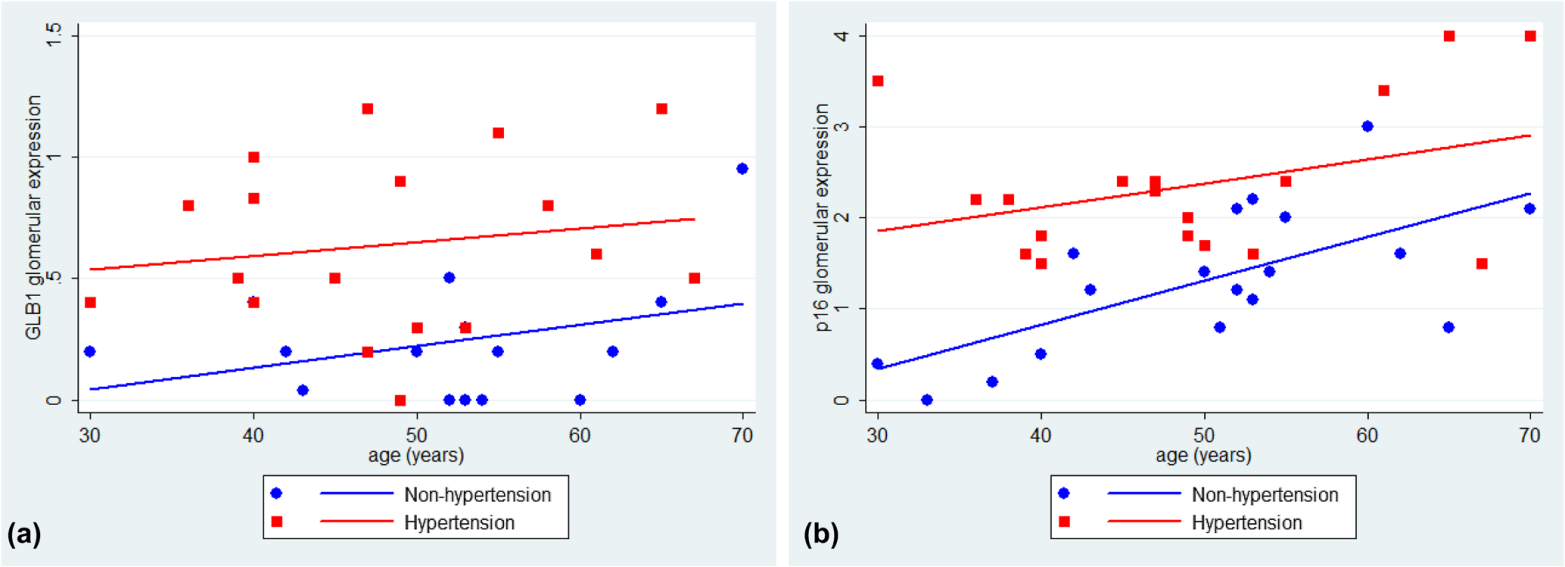
(a) Linear prediction of age and glomerular GLB1 expression in hypertensive and non‐hypertensive kidneys. The glomerular expression of the senescent marker GLB1 is significantly related to hypertension (*p* < 0.001) but not age (*p* = 0.20). (b) Linear prediction of age and p16 expression in hypertensive and non‐hypertensive kidneys. The glomerular expression of p16 is significantly related to hypertension (*p* < 0.001). In non‐hypertensive kidneys, glomerular p16 expression is significantly related to age (*p* = 0.003).

Hypertensive patients were age‐matched with non‐hypertensive patients in the age range of 30–70 years old. In both hypertensive and non‐hypertensive subjects, p16 and GLB1 were identified in glomeruli during TC, and glomerular expression of p16 and GLB1 was significantly increased in hypertension (Figure 5). In linear regression analysis, glomerular p16 expression was significantly correlated with age in non‐hypertensive subjects (coefficient 9.140, 95% CI 3.676–14.603, *p* = 0.003) but not in patients with hypertension (coefficient 4.865, 95% CI −1.868 to 11.598, *p* = 0.15).

## 4 DISCUSSION

FSGS in hypertensive and non‐hypertensive kidneys develops through a process that begins with glomerular TC and capsular fibrosis. In studies by ourselves and others, the average volumes of “normal” glomeruli in hypertensive kidneys were found to be larger than those in non‐hypertensive kidneys (Denic et al., 2018; Hughson et al., 2014; Puelles et al., 2016). In this study, we found considerable overlap in the volumes of these “normal” glomeruli in hypertensive and non‐hypertensive subjects, particularly in the mid and lower quartiles of glomerular size, with hypertension being skewed toward very large volumes. There was a significant direct correlation between larger glomerular size and a higher proportion of GGS, suggesting that larger volumes are an adaptation to nephron loss rather than a primary attribute of hypertension (Denic, Lieske, et al., 2017; Denic, Mathew, et al., 2017; Hommos et al., 2017; Hughson et al., 2014).

The mechanisms leading to FGGS have been discussed for decades. The conventional opinion is that nephrosclerosis results from hypertension, which first affects the intrarenal arteries and, secondarily, leads to glomerulosclerosis as a result of reduced perfusion through arteriosclerotic preglomerular blood vessels (Hommos et al., 2017; Hughson et al., 2014; Kopp, 2013). As people age, hypertension becomes more prevalent. Depending on race, geography, and socioeconomic status, hypertension affects 70% of many populations by 60 years of age (Ostchega et al., 2020; Klag et al., 1996; Kopp, 2013). Because of its association with aging, hypertension is proposed to be a disease of biological senescence, and Shankland et al. (2023) and Hodgin et al. (2015) suggest that podocyte loss due to senescence rather than arteriosclerosis underlies the development of FGGS.

The science of senescence is relatively new. Previously, senescence was thought to affect dividing cells that had lost their reproductive capacity due to telomerase depletion (Gonzales‐Gualda et al., 2021). Currently, replicative is distinguished from non‐replicative senescence (Shankland et al., 2023). Non‐replicative senescence best fits models of podocyte pathology, as podocytes are considered nondividing cells that live as long as the kidney.

Immunohistochemical biomarkers of senescence include increased expression of p16 and p21. Both are cyclin‐dependent kinase inhibitors of the retinoblastoma protein and are associated with p53 expression (Gonzales‐Gualda et al., 2021). The tumor suppressor p53 induces the transcription of p21 in response to DNA damage. Cell cycle inhibition by p21 enables p53 to promote apoptosis and eliminate damaged cells. In contrast, p16 directly inhibits the function of the retinoblastoma protein. p16 can promote p53 degradation through the proteasomal pathway, and with aging, increased p16 levels can reduce p53 levels and the cell's ability to respond to DNA damage. In our hypertensive biopsies, some variably intense p53 expression, consistent with wild‐type p53, was observed in tubules; however, no p53 staining was found in “normal” glomeruli or glomeruli undergoing TC. Glomerular p16 and p21 were increased with TC, with the glomerular p16 expression being limited to PECs. The findings suggest a role for p16 in FGGS associated with PEC rather than podocyte senescence. There is a significant increase in tubular p16 and p21 in hypertension, suggesting that both cell cycle inhibitors contribute to tubular atrophy, independent of factors that lead to glomerulosclerosis.

Many in the field regard β‐galactosidase as a senescence‐specific biomarker, particularly the isoform active at pH 6 (Abe & Shayman, 2013; Gonzales‐Gualda et al., 2021). Lysosomal pH 6‐specific β‐galactosidase degrades cellular elements during autophagocytosis and identifies cells having a “secretory phenotype,” with secretory referring to the paracrine release of inflammatory cytokines, growth regulators, and factors affecting the extracellular matrix (Gonzales‐Gualda et al., 2021; Shankland et al., 2023). We describe senescent biomarkers in hypertensive and non‐hypertensive kidneys in tubules and arteries, but for this study, we emphasize senescence markers in glomeruli during the development of FGGS.

β‐galactosidase was found in contracting glomeruli within cells consistent with podocytes and PECs. The antibody used in our immunohistochemistry reacts with GLB1 β‐galactosidase, a lysosomal enzyme active at an acidic pH (Abe & Shayman, 2013). Glomeruli undergoing TC also expressed 5′‐nucleotidase (CD73) in a distribution similar to β‐galactosidase. CD73 is not considered a senescence marker but rather a response to oxidative stress that modulates inflammation and thrombosis by the generation and breakdown of ATP, ADP, and AMP (Alcedo et al., 2021). CD73 is found intrinsically on most interstitial but not glomerular endothelium and becomes secondarily expressed on podocytes with proteinuria (Popovic et al., 2021). A relationship between CD73 and arteriosclerosis, as a possible senescence factor, has recently been proposed (Sutton et al., 2020).

The senescence biomarkers in glomeruli undergoing TC coexisted with PEC activation. During TC, hypertrophy and increased PEC numbers were identified, with activation supported by their positive reactions for CK7 and CD44. It is well established that CD44 signaling regulates cellular proliferation and migration (Eymael et al., 2018; Roeder et al., 2017). Our study revealed a decline in podocyte numbers with TC, suggesting that TC and PEC activation were related to podocyte loss. It is worth noting that CD44 and CK7 also serve as markers of epithelial‐to‐mesenchymal transition (Eymael et al., 2018; Roeder et al., 2017), which may support the contribution of activated PECs to intracapsular collagenization, whose cellular origin in FGGS remains unclear.

The activation of PECs increases their potential for migration, proliferation, and extracellular matrix production (Eymael et al., 2018) and constitutes one of the pathogenetic mechanisms underlying FSGS, a primary pattern of glomerular pathology often confused with FGGS (Kuppe et al., 2015, 2019; Wiggins, 2007). In FSGS, PECs are activated after podocyte depletion and possibly by immunological stimuli. Importantly, the mechanisms driving PECs to contribute to different pathological patterns remain unclear (Eymael et al., 2018; Kuppe et al., 2015, 2019; Lazareth et al., 2019; Wiggins, 2007).

CD44 activation of PECs has been described in aging mice with glomerulosclerosis that resembles FSGS rather than FGGS (Hamatani et al., 2020). To the best of our knowledge, our current study is the first to describe PEC activation in the FGGS of human aging and hypertension. During TC in hypertensive patients, PEC activation is not associated with the segmental capsular adhesions that are clearly shown to follow podocyte injury in FSGS (Eymael et al., 2018; Kuppe et al., 2015, 2019; Smeets et al., 2006). The absence of segmental lesions during TC and intracapsular fibrosis suggests that podocyte‐PEC interactions in FGGS are fundamentally unique. The contraction phase of FGGS is characterized by PEC activation that becomes surrounded by acellular connective tissue, with little, if any, contact between the tuft and the basement membrane of the Bowman's capsule. This envelopment may account for the slow progression of “benign” hypertension compared to the destructive features of primary sclerosing glomerular diseases (Hughson et al., 2014).

There is an absence of senescence markers in the “normal” glomeruli of hypertensive patients. There is also an absence of senescent markers in “normal” post‐transplant kidneys, where adverse stimuli might be expected. In this regard, the standard IHC methods used for this study may not be adequately sensitive to detect early senescence that may precede any obvious pathology. Nevertheless, in FGGS, the expression of senescence markers is associated with the development of intracapsular fibrosis that sequesters the contracting tuft from Bowman's capsule and appears to mitigate against the development of segmental sclerosis lesions.

## AUTHOR CONTRIBUTIONS

MDH, AAA, VGP, and JFB equally contributed to the study design. AAA was responsible for collecting case material, clinical histories, and laboratory data. MDH and AAA contributed equally to the immunohistochemistry for senescent markers, glomerular size measurements, and podocyte counting. VGP and YO performed the testing and analysis of activation markers. All authors contributed to the analyses of the findings. MDH wrote the first manuscript draft. All authors contributed to revisions and approved the final manuscript.

## FUNDING INFORMATION

No funding information provided.

## CONFLICT OF INTEREST STATEMENT

All authors have no competing interests.

## ETHICS STATEMENT

The research used human tissue archived as paraffin blocks in Departmental files and was performed in compliance with the Helsinki Accords. The Scientific Research Unit of Hiwa Hematology and Oncology serves as the regional Institutional Review Board approved the research as exempt from informed consent.

## Supporting information


Data S1.

## Data Availability

Biopsy data are stored in Excel files and are submitted as Data S1.

## References

[phy270730-bib-0001] Abe, A. , & Shayman, J. A. (2013). Sphingolipid catabolism. In W. J. Lennarz & D. Lane (Eds.), Encyclopedia of Biological Chemistry (2nd ed., pp. 287–292). Elsevier, Inc.

[phy270730-bib-0002] Alcedo, K. P. , Bowser, J. L. , & Snider, N. T. (2021). The elegant complexity of mammalian ecto‐5‐nucleotidase (CD73). Trends in Cell Biology, 31, 829–842. 34116887 10.1016/j.tcb.2021.05.008 PMC8448938

[phy270730-bib-0003] Burrows, N. R. , Koyama, A. , & Pavkov, M. E. (2022). Reported cases of end‐stage kidney disease‐United States, 2000‐2019. MMWR. Morbidity and Mortality Weekly Report, 71, 412–415. 35298452 10.15585/mmwr.mm7111a3 PMC8942306

[phy270730-bib-0004] Denic, A. , Lieske, J. C. , Chakkera, H. A. , Poggio, E. D. , Alexander, M. P. , Singh, P. , Kremers, W. K. , Lerman, L. O. , & Rule, A. D. (2017). The substantial loss of nephrons in healthy human kidneys with aging. Journal of the American Society of Nephrology, 28, 313–320. 27401688 10.1681/ASN.2016020154 PMC5198286

[phy270730-bib-0005] Denic, A. , Mathew, J. , Lerman, L. O. , Lieske, J. C. , Larson, J. J. , Alexander, M. P. , Poggio, E. , Glassock, R. J. , & Rule, A. D. (2017). Single‐nephron glomerular filtration rate in healthy adults. The New England Journal of Medicine, 376, 2349–2357. 28614683 10.1056/NEJMoa1614329 PMC5664219

[phy270730-bib-0006] Denic, A. , Mathew, J. , Nagineni, V. V. , Thompson, R. H. , Leibovich, B. C. , Lerman, L. O. , Lieske, J. C. , Alexander, M. P. , Augustine, J. J. , Kremers, W. K. , & Rule, A. D. (2018). Clinical and pathological findings associate consistently with larger glomerular volume. Journal of the American Society of Nephrology, 29, 1960–1969. 29789431 10.1681/ASN.2017121305 PMC6050922

[phy270730-bib-0007] Eymael, J. , Sharma, S. , Loeven, M. A. , Wetzels, J. F. , Mooren, F. , Florquin, S. , Deegans, J. K. , Willemsen, B. K. , Sharma, V. , van Kuppevelt, T. H. , Bakker, M. A. , Ostendorf, T. , Moeller, M. J. , Dijkman, H. B. , Smeets, B. , & van der Vlag, J. (2018). CD44 is required for the pathogenesis of experimental crescentic glomerulonephritis and collapsing focal segmental glomerulosclerosis. Kidney International, 93, 626–642. 29276101 10.1016/j.kint.2017.09.020

[phy270730-bib-0008] Gonzales‐Gualda, E. , Baker, A. G. , Fruk, L. , & Munoz‐Espin, B. (2021). A guide to assessing cellular senescence in vitro and in vivo. FEBS Journal, 228, 56–80. 10.1111/febs.15570 32961620

[phy270730-bib-0009] Hall, J. E. , Granger, J. P. , do Carmo, J. M. , da Silva, A. A. , Dubinion, J. , George, E. , Hamza, S. , Speed, J. , & Hall, M. E. (2012). Hypertension: Physiology and pathophysiology. Comprehensive Physiology, 2, 2393–2442. 23720252 10.1002/cphy.c110058

[phy270730-bib-0010] Hamatani, H. , Eng, D. G. , Hiromura, K. , Pippin, J. W. , & Shankland, S. J. (2020). CD44 impacts glomerular parietal epithelial cell changes in the aged mouse kidney. Physiological Reports, 8, e14487. 32597007 10.14814/phy2.14487 PMC7322268

[phy270730-bib-0011] Hodgin, J. B. , Bitzer, M. , Wickman, L. , Afshinnia, F. , Wang, S. Q. , O'Conner, C. , Yang, Y. , Meadowbrooke, C. , Chowdhury, M. , Kikuchi, M. , Wiggins, J. E. , & Wiggins, R. C. (2015). Glomerular aging and focal global glomerulosclerosis: A podometric perspective. Journal of the American Society of Nephrology, 26, 3162–3178. 26038526 10.1681/ASN.2014080752 PMC4657829

[phy270730-bib-0012] Hommos, M. S. , Glassock, R. J. , & Rule, A. D. (2017). Structural and functional changes in human kidneys with healthy aging. Journal of the American Society of Nephrology, 28, 2838–2844. 28790143 10.1681/ASN.2017040421 PMC5619977

[phy270730-bib-0013] Hughson, M. , Farris, A. B., III , Douglass‐Denton, R. , Hoy, W. E. , & Bertram, J. F. (2003). Glomerular number and size in autopsy kidneys: The relationship to birth weight. Kidney International, 63, 2113–2122. 12753298 10.1046/j.1523-1755.2003.00018.x

[phy270730-bib-0014] Hughson, M. D. , Johnson, K. , Young, R. , Hoy, W. E. , & Bertram, J. F. (2002). Glomerular size and glomerulosclerosis: Relationships to disease categories, glomerular solidification, and ischemic obsolescence. American Journal of Kidney Diseases, 39, 679–688. 11920332 10.1053/ajkd.2002.31980

[phy270730-bib-0015] Hughson, M. D. , Puelles, V. G. , Hoy, W. E. , Duglas‐Denton, R. N. , Mott, S. A. , & Bertram, J. F. (2014). Hypertension, glomerular hypertrophy, and nephrosclerosis: The effect of race. Nephrology, Dialysis, Transplantation, 29, 1399–1409. 10.1093/ndt/gft480 PMC4071048 24327566

[phy270730-bib-0016] Klag, M. I. , Whelton, P. K. , Randall, B. L. , Neaton, J. D. , Brancati, F. L. , Ford, C. E. , Shulman, N. B. , & Stamler, J. (1996). Blood pressure and end‐stage renal disease in men. The New England Journal of Medicine, 334, 13–18. 7494564 10.1056/NEJM199601043340103

[phy270730-bib-0017] Kopp, J. B. (2013). Rethinking hypertensive kidney disease: Arterionephrosclerosis as a genetic, metabolic, and inflammatory disorder. Current Opinion in Nephrology and Hypertension, 22, 266–272. 23470819 10.1097/MNH.0b013e3283600f8c PMC4165431

[phy270730-bib-0018] Kuppe, C. , Grone, H.‐J. , Ostendorf, T. , van Kuppevelt, T. H. , Boor, P. , Floege, J. , Smeets, B. , & Moeller, M. J. (2015). Common histologic patterns in glomerular epithelial cells in secondary focal segmental glomerulosclerosis. Kidney International, 88, 990–998. 25853334 10.1038/ki.2015.116

[phy270730-bib-0019] Kuppe, C. , Leuctle, K. , Wagner, A. , Kabgani, N. , Saritas, T. , Puelles, V. G. , Smeets, B. , Hakroush, S. , van der Vlag, J. , Boor, P. , Schiffer, M. , Grone, H.‐J. , Fogo, A. , Floege, J. , & Moeller, M. J. (2019). Novel parietal epithelial cell subpopulations contribute to focal segmental glomerulosclerosis and glomerular tip lesions. Kidney International, 96, 80–93. 31029503 10.1016/j.kint.2019.01.037 PMC7292612

[phy270730-bib-0020] Lazareth, H. , Henique, C. , Lenoir, C. , Puelles, V. G. , Flamant, M. , Bolee, G. , Fligny, C. , Camus, M. , Guyonnet, L. , Millien, C. , Gaillard, F. , Chipont, A. , Robin, B. , Fabrega, S. , Dhaun, N. , Camerer, E. , Kretz, O. , Grahammer, F. , Braun, F. , … Tharaux, P.‐L. (2019). The tetraspanin CD9 controls migration and proliferation of parietal epithelial cells and glomerular disease progression. Nature Communications, 10, 3303. 10.1038/s41467-019-11013-2 PMC6656772 31341160

[phy270730-bib-0021] Ostchega, Y. , Fryar, C. D. , Nwankwo, T. , & Nguyen, D. T. (2020). Hypertension prevalence among adults 18 and over: United States, 2017–2018. NCHS Data Brief. N0. 364. April 2020. Centers for Disease Control and Prevention. National Center for Health Statistics. U.S. Department of Health and Human Services.

[phy270730-bib-0022] Popovic, Z. V. , Bestvater, F. , Krunic, D. , Kramer, B. K. , Bergner, R. , Lufter, C. , Hoher, B. , Marx, A. , & Purubsky, S. (2021). CD73 overexpression in podocytes: A novel marker of podocyte injury in human kidney disease. International Journal of Molecular Sciences, 22, 7642. 34299260 10.3390/ijms22147642 PMC8304086

[phy270730-bib-0023] Puelles, V. G. , Cullen‐McEwen, L. A. , Taylor, G. E. , Li, J. , Hughson, M. D. , Kerr, P. G. , Hoy, W. E. , & Bertram, J. F. (2016). Human podocyte depletion in association with older age and hypertension. American Journal of Physiology. Renal Physiology, 310, F656–F668. 26792066 10.1152/ajprenal.00497.2015

[phy270730-bib-0024] Roeder, S. S. , Barnes, T. J. , Lee, J. S. , Kato, I. , Eng, D. G. , Kaverina, N. V. , Sunseri, M. W. , Daniel, C. , Amann, K. , Pippin, J. W. , & Shankland, S. J. (2017). Activated ERK1/2 increases CD44 in glomerular epithelial cells leading to matrix expansion. Kidney International, 91, 896–913. 27998643 10.1016/j.kint.2016.10.015 PMC5357449

[phy270730-bib-0025] Shankland, S. J. , Rule, A. D. , Kutz, J. N. , Pippin, J. W. , & Wessely, O. (2023). Podocyte senescence and aging. Kidney360, 4, 1784–1793. 37950369 10.34067/KID.0000000000000284 PMC10758523

[phy270730-bib-0026] Smeets, B. , Dijkman, H. B. P. M. , Wetzels, J. F. M. , & Steenbergen, E. J. (2006). Lessons from studies on focal segmental glomerulosclerosis: An important role for parietal epithelial cells? The Journal of Pathology, 210, 263–272. 16924588 10.1002/path.2051

[phy270730-bib-0027] Sutton, N. R. , Bouis, D. , Mann, K. M. , Rashid, M. , McCubberey, A. L. , Hyman, M. C. , Goldstein, D. R. , Mei, A. , & Pinsky, D. J. (2020). CD73 promotes age‐dependent accretion of atherosclerosis. Arteriosclerosis, Thrombosis, and Vascular Biology, 40, 61–71. 31619062 10.1161/ATVBAHA.119.313002 PMC7956240

[phy270730-bib-0028] Venkatareddy, M. , Wang, S. , Yang, Y. , Patel, S. , Wickman, L. , Nishizon, R. , Chowdhury, M. , Hodgin, J. , Wiggins, P. A. , & Wiggins, R. C. (2014). Estimating podocyte number and density using a single histologic section. Journal of the American Society of Nephrology, 25, 1116–1129. 10.1681/ASN.2013080859 PMC4005315 24357669

[phy270730-bib-0029] Wang, G. , Lai, F. M. , Kwan, B. C. , Lai, K. B. , Chow, K. M. , Li, P. K. , & Szetp, C. C. (2009). Podocyte loss in human hypertensive nephrosclerosis. American Journal of Hypertension, 22, 300–306. 19131934 10.1038/ajh.2008.360

[phy270730-bib-0030] Weibel, E. R. , & Gomez, D. M. (1962). A principle for counting tissue structures on random sections. Journal of Applied Physiology (1985), 17, 343–348. 10.1152/jappl.1962.17.2.343 14005589

[phy270730-bib-0031] Wiggins, R. C. (2007). The spectrum of podocytopathies: A unifying view of glomerular diseases. Kidney International, 71, 1205–1214. 17410103 10.1038/sj.ki.5002222

